# Hormonal regulation and reproductive improvement with adjunctive Zishen Yutai Pill in polycystic ovary syndrome: a systematic review with meta-analysis

**DOI:** 10.3389/frph.2025.1748768

**Published:** 2026-01-15

**Authors:** Xijing Lu, Enoch Chi Ngai Lim, Genping Zeng, Chi Eung Danforn Lim, Lei Zeng

**Affiliations:** 1 The First Affiliated Hospital of Guangzhou University of Chinese Medicine, Guangzhou, Guangdong Province, China; 2 First Clinical School of Medicine of the Guangzhou University of Chinese Medicine, Guangzhou, Guangdong Province, China; 3 Translational Research Department, Specialist Medical Services Group, Earlwood, NSW, Australia; 4 School of Life Sciences, University of Technology Sydney, Ultimo, NSW, Australia; 5 NICM Health Research Institute, Western Sydney University, Westmead, NSW, Australia

**Keywords:** Zishen Yutai Pill, polycystic ovary syndrome, PCOS, Chinese patent medicine, systematic review, meta-analysis

## Abstract

**Objective:**

To systematically evaluate the therapeutic efficacy and safety of Zishen Yutai Pill (ZYP) as an adjunctive treatment for women with polycystic ovary syndrome (PCOS) through systematic review and meta-analysis of randomized controlled trials.

**Materials and methods:**

A systematic review and meta-analysis were conducted in accordance with PRISMA guidelines. Seven major databases, including PubMed, Embase, Web of Science, Cochrane Library, CNKI, VIP, and Wan Fang Database, were searched from inception through March 1, 2025. Randomized controlled trials comparing ZYP combined with Western medicines vs. Western medicines alone were included.

**Results:**

Eighteen randomized controlled trials encompassing 1,751 participants with PCOS met the inclusion criteria. Meta-analysis demonstrated that ZYP, when combined with Western medicines, produced statistically significant improvements compared with Western medicines alone. Combination therapy significantly enhanced pregnancy rates [relative risk [RR] = 1.52, 95% confidence interval [CI] = 1.35–1.76, *P* < 0.00001] and ovulation rates (RR = 1.20, 95% CI = 1.12–1.30, *P* < 0.00001). ZYP combination therapy significantly increased endometrial thickness [mean difference (MD) = 1.34, 95% CI = 1.03–1.65, *P* < 0.00001]. Hormonal analysis revealed significant reductions in testosterone levels [standard mean difference (SMD) = −1.90, 95% CI = −2.94 to −0.86, *P* = 0.0003] and luteinizing hormone levels (SMD = −0.77, 95% CI = −1.21 to −0.33, *P* = 0.0006). Combination therapy significantly reduced miscarriage rates (RR = 0.54, 95% CI = 0.40–0.72, *P* < 0.0001).

**Conclusion:**

This systematic review and meta-analysis suggests that adjunctive ZYP combined with Western medicines may improve reproductive outcomes in women with PCOS. However, the certainty of evidence for most outcomes was low or very low, and many trials had high or unclear risk of bias. Accordingly, these findings should be interpreted as hypothesis-supporting rather than practice-changing, and well-designed, independently funded multicenter randomized controlled trials with standardized outcome definitions are required before routine clinical use or guideline integration can be considered.

**Systematic Review Registration:**

PROSPERO CRD42024522660.

## 1 Introduction

Polycystic ovary syndrome (PCOS) represents one of the most prevalent reproductive endocrine disorders affecting women of reproductive age, with prevalence rates ranging from 5% to 18% in this population (1, 2). This condition imposes substantial economic and societal burdens (3), manifesting as a systemic and heterogeneous disorder characterized by sporadic ovulation or anovulation, polycystic ovary morphology (PCOM), and clinical or biochemical signs of hyperandrogenism (HA), including dysmenorrhea, hirsutism, acne, obesity, and infertility.

PCOS is frequently associated with endocrine and metabolic abnormalities, leading to ovulatory dysfunction, which constitutes a primary cause of both primary and secondary infertility in affected women (4). Approximately 80% of women with PCOS experience infertility due to ovulatory dysfunction (5). The underlying mechanisms may involve overproduction of ovarian androgens, insulin resistance (IR) resulting in hyperinsulinemia, obesity, and disrupted paracrine signalling within the ovary (6, 7).

Current therapeutic approaches for PCOS-related infertility primarily focus on ovulation induction therapy to increase conception rates (8). The 2023 International Evidence-based Guideline for the Assessment and Management of Polycystic Ovary Syndrome recommends letrozole, clomiphene, metformin, and combined clomiphene-metformin therapy as first-line treatments. Second-line interventions include gonadotropins or laparoscopic ovarian drilling, while *in vitro* Fertilization-Embryo Transfer (IVF-ET) serves as a third-line option (9).

However, conventional therapies present significant limitations and risks. Letrozole has been associated with spontaneous abortions and congenital disabilities (10), while treatments for PCOS accompanied by metabolic syndrome may increase obstetric complication risks (11). This concern is particularly relevant given that PCOS patients face an increased risk of metabolic disorders, including metabolic syndrome and insulin resistance (12). Other ovulation stimulants such as clomiphene and gonadotropins may lead to ovarian hyperstimulation syndrome and multiple pregnancies (13, 14).

Given these limitations and side effects of conventional therapies, many PCOS patients seek complementary and alternative medicines (CAMs), with growing evidence supporting their use in mitigating PCOS severity and associated complications (15, 16). Surveys indicate that a significant proportion of women utilize herbal CAMs, particularly when facing fertility challenges (17, 18).

Chinese herbal medicine, specifically the Zishen Yutai Pill (ZYP), represents a proprietary blend of various Chinese herbs that shows promise in treating reproductive and metabolic defects in PCOS (19). Research indicates that ZYP can increase estradiol (E2) and progesterone levels, reduce luteinizing hormone (LH) and testosterone (T) levels, and promote follicle development and ovulation (20). Multiple studies have demonstrated that ZYP can increase the number of high-quality embryos, repair the endometrium, improve uterine receptivity, and treat luteal phase defects, menstrual disorders, and ovarian dysfunction (21). Clinical trials suggest that ZYP, particularly when combined with Western medicines, improves ovulation and pregnancy rates as well as early pregnancy outcomes more effectively than Western medicines alone (22).

Despite promising findings, existing ZYP studies are limited by small sample sizes and varied control groups. To address this gap, this study is the first systematic review and meta-analysis of randomized controlled trials (RCTs) evaluating ZYP efficacy, particularly in combination with Western medicines, for the treatment of PCOS.

## 2 Methods

This systematic review was conducted in strict accordance with the Cochrane Handbook for Systematic Reviews and PRISMA guidelines. The study was registered with PROSPERO (registration ID: CRD42024522660).

### 2.1 Search strategy

PubMed, Embase, Web of Science, Cochrane Library, China National Knowledge Infrastructure (CNKI), China Science and Technology Journal Database (VIP Database), and Wan Fang Database were searched from inception to March 1, 2025. Search terms included variations of “Zishen Yutai Pill” and “polycystic ovary syndrome” across English and Chinese databases. Detailed search strategies for each database are provided in Supplementary Table 1. Additional sources, including ClinicalTrials.gov, the Chinese Clinical Trial Registry, and grey literature databases such as Opengrey and Greyguide, were searched to identify unpublished studies and minimize publication bias.

### 2.2 Selection criteria

#### 2.2.1 Inclusion criteria

Studies met the following inclusion criteria: (1) patients meeting PCOS diagnostic criteria, including menstrual abnormalities (sporadic ovulation or anovulation), clinical manifestations of hyperandrogenism and/or biochemical hyperandrogenism, and polycystic ovary morphology (PCOM), with exclusion of other disorders causing ovulation disorders (thyroid dysfunction, premature ovarian failure, hypothalamic-pituitary amenorrhea, hyperprolactinemia) and hyperandrogenemia (Cushing's syndrome, atypical adrenal-genital syndrome, androgen-secreting endocrine tumors); (2) randomized controlled trial design; (3) comparison of ZYP plus Western medicines vs. Western medicines alone, with identical Western medicines used in both test and control groups, including menstrual cycle regulation agents, ovulation induction agents, and dydrogesterone; (4) PCOS patients achieving natural conception; (5) inclusion of at least one outcome measure: ovulation rate, pregnancy rate, or sex hormone indices (LH, E2, T, FSH).

#### 2.2.2 Exclusion criteria

Studies were excluded if they met any of the following criteria: (1) non-randomized controlled trials, reviews, meta-analyses, cohort studies, case reports, basic science papers, study protocols, literature reviews, and animal experiments; (2) control groups utilizing ZYP; (3) experimental groups employing Chinese medicine decoctions and patent prescriptions other than ZYP, as well as Chinese medicine (CM) treatments such as acupuncture, massage, qigong, Tai Chi, fire cupping, and moxibustion as co-interventions; (4) PCOS patients conceived through assisted reproductive techniques such as artificial insemination (AI) or IVF-ET; (5) duplicate studies.

### 2.3 Outcomes

Primary outcomes included ovulation and pregnancy rates. Secondary outcomes encompassed miscarriage rate, sex hormones (FSH, LH, T, E2), and endometrial thickness.

Pregnancy rate was defined as the proportion of participants achieving pregnancy during the study period. Definitions of pregnancy varied across included trials: most studies reported ultrasound-confirmed intrauterine pregnancy (clinical pregnancy), one study defined pregnancy based on a positive urine pregnancy test, and four studies did not explicitly specify the pregnancy definition. For studies without a clearly stated definition, pregnancy outcomes were extracted as reported. Given the variability in outcome definitions, additional sensitivity analyses were conducted to assess the robustness of the pooled estimates.

### 2.4 Literature selection

Studies were managed using EndNote X9 for duplicate removal. Two authors independently screened titles and abstracts, with full texts reviewed for final inclusion. Discrepancies were resolved through discussion or consultation with a third reviewer when necessary. The process was documented via a PRISMA flowchart.

### 2.5 Data extraction

Study characteristics were meticulously extracted, including first author, publication year, patient numbers in experimental and control groups, mean age, experimental group intervention, control group intervention, intervention duration, drug name, and outcomes. For studies utilizing treatment regimens involving two or more sequential phases, only outcome data collected after first-phase intervention were extracted. When sufficient information was unavailable from publications, trial protocols, clinical study reports, or other sources, attempts were made to contact the study authors to obtain missing data for risk-of-bias assessment.

### 2.6 Assessment of risk of bias

The risk of bias (RoB) for each outcome was independently assessed by two authors using the Cochrane RoB 2 method, as recommended in the Cochrane Handbook (version 6.4). Discrepancies in assessment were resolved through negotiation or consultation with a third author. When missing data could not be acquired within a specified timeframe or from the study authors, the impact of such missing data on the stability of the results was assessed through sensitivity analyses. Subjective evaluations were excluded unless explicitly stated. Assumptions and extrapolations regarding missing data were clearly described, and sensitivity analyses were conducted to assess the impact of these extrapolations. Desired key data were calculated from other statistics in accordance with the Cochrane Handbook guidelines.

Bias risk assessment encompasses multiple domains, including bias arising from randomization, deviations from intended interventions, missing outcome data, outcome measurement, and selection of reported results. Each bias domain for each outcome was classified as high, unclear, or low risk, with overall risk of bias determined for each outcome as: (i) low risk, with all domains judged low risk; (ii) unclear risk, with one or more domains judged unclear risk; and (iii) high risk, with one or more domains judged high risk or four domains judged unclear risk. RevMan 5.4.1 from the Cochrane Collaboration was used to display results.

Publication bias was evaluated using STATA 15.1 via funnel plot visualization and Egger's linear regression test. Funnel plots were constructed with effect size on the horizontal axis and standard error on the vertical axis, with visual inspection used to assess symmetry. Egger's test quantified funnel plot asymmetry through a regression model, with a significance threshold of *P* < 0.05 indicating potential publication bias. For outcomes with fewer than 10 included studies, given the limited statistical power, Egger's test results were interpreted cautiously, and publication bias was primarily evaluated through a visual assessment of funnel plots for symmetry. When bias risk was detected, the Trim and Fill method was applied to estimate the impact of missing studies and adjust the pooled effect size accordingly. To enhance clarity and readability, detailed meta-regression outputs and subgroup analyses are presented in the Supplementary Materials, while the main text focuses on clinically relevant effect estimates.

### 2.7 Statistical analysis

A meta-analysis was conducted using RevMan 5.4.1, with statistical significance defined as a two-sided *P* < 0.05. For dichotomous variables, pooled risk ratios (RRs) with 95% confidence intervals (CIs) were calculated as the primary effect measure. RR was selected over the odds ratio (OR) because it is more directly interpretable for common reproductive outcomes, whereas OR may overestimate effects (23). For continuous outcomes, mean differences (MDs) with 95% confidence intervals (CIs) were pooled directly when all studies reported outcomes using consistent measurement units and scales. When studies employed heterogeneous measurement instruments or units, standardized mean differences (SMDs) with 95% CIs were calculated to harmonize effect sizes across studies. After examining baseline change and endpoint data separately, mean and standard deviation data at endpoint for both intervention and comparison groups were extracted if the included studies did not report baseline changes. When studies included both baseline changes and endpoint data, unstandardized means were utilized and combined in subsequent meta-analyses.

Heterogeneity tests were conducted using the Q-value statistic and the *I*^2^ test. A fixed-effects model was employed if *P* > 0.10 and *I*^2^ ≤ 50%, indicating no statistical heterogeneity between studies. Conversely, a random-effects model was utilized if *P* ≤ 0.10 and *I*^2^ > 50%, indicating statistical heterogeneity. STATA 15.1 and RevMan 5.4.1 were employed to analyze heterogeneity sources exploratorily. *A priori* covariables considered included age, infertility duration, treatment course, comorbid infertility status, syndrome-based medication use, ZYP administration method, baseline indicator levels, region, and Western medicine type. Although Body Mass Index (BMI) was initially prespecified due to its known role as a potential effect modifier in PCOS, insufficient and inconsistently reported BMI data across trials precluded its inclusion in meta-regression or subgroup analyses.

Univariate meta-regression was first used to preliminarily screen significant covariables (*P* < 0.1). To avoid overfitting, the number of covariables included in multivariate meta-regression was strictly limited by calculating variance inflation factors (VIF < 5) to ensure no strong collinearity and combining with the clinical importance of covariables. After identifying independent predictors via multivariate meta-regression, subgroup analyses were further conducted to validate group differences. A covariable was considered a potential heterogeneity source if: (1) Cochran's *Q* test *P*-value across subgroups was < 0.05; (2) *I*^2^ within each subgroup significantly decreased after stratification; and (3) effect sizes across subgroups showed consistent directions and clinically meaningful magnitude differences.

Sensitivity analyses were conducted using STATA 15.1 to assess the robustness of the results and identify potential sources of heterogeneity. Specifically, leave-one-out analysis was performed by sequentially excluding each study and recalculating pooled effect estimates. Clinical heterogeneity was evaluated through subgroup analyses and qualitative assessment of differences in study populations, interventions, and outcome measurements. Summary tables of results were created using GRADEPro 3.6.1 to evaluate evidence quality and provide evidence-based recommendations for this systematic review.

## 3 Results

### 3.1 Screening results

**Figure 1:**
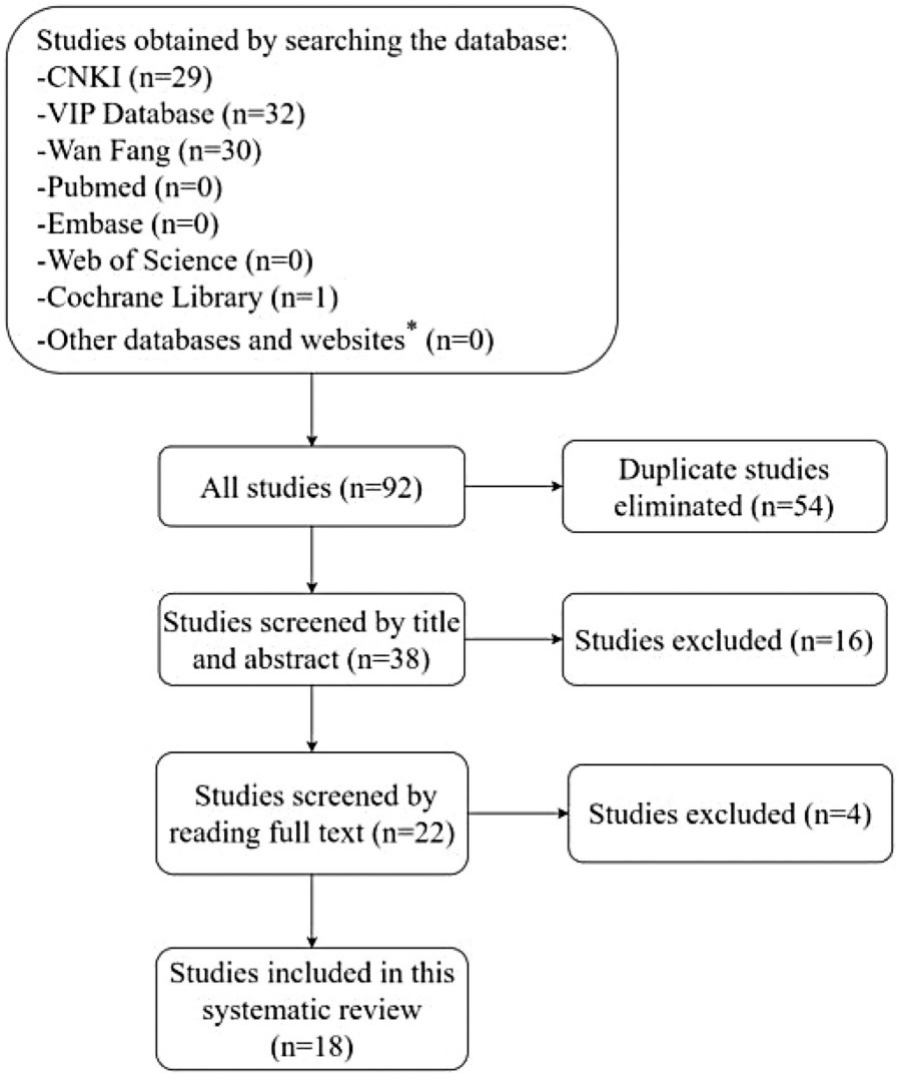
Flow diagram depicting the selection of studies included in the systematic review. This diagram illustrates the comprehensive search and selection process, from initial article retrieval to final study inclusion. *Other databases and websites include ClinicalTrials.gov, the Chinese Clinical Trial registry and grey literature databases such as Opengrey and Greyguide.

The search of seven databases yielded 1,751 articles. After removing duplicates and applying inclusion and exclusion criteria, 18 RCTs (22, 24–40) met the selection criteria and were included in the final analysis (Figure 1).

### 3.2 Study characteristics

The 18 included studies (22, 24–40) were published between 2008 and 2024, encompassing 1,751 participants with PCOS. Sample sizes ranged from 60 to 180 participants across studies. All studies compared ZYP combined with Western medicines vs. Western medicines alone. Western medicines included ovulation induction agents (letrozole, clomiphene citrate), menstrual cycle regulators (ethinylestradiol and cyproterone acetate tablets, Diane-35, Yasmin), and luteal support agents (dydrogesterone). Treatment durations varied from 1 to 6 months to 3 menstrual cycles. Study populations were predominantly from China, with participants' ages ranging from 20 to 35 years. The characteristics of the included studies are summarized in Table 1.

**Table 1:** Characteristics of the included studies.

Author/Year	Groups	Sample size	Mean age (year)	Intervention measures	Diagnostic criteria used	Interven-tion times (menstrual cycles)	Outcome index
Zhang (2022) (40)	EG	49	28.16 ± 2.70	LE + DDG + ZYP	2018 Chinese guideline	3	Pregnancy rate
	CG	48	28.23 ± 2.55	LE + DDG		3	
Hong, (2016) (24)	EG	67	28.42 ± 2.87	CC + ZYP	Rotterdam criteria	3	Pregnancy rate; Ovulation rate; Miscarriage rate
	CG	67	27.95 ± 3.21	CC		3	
Li (2019) (31)	EG	50	27.86 ± 6.35	Diane-35 + ZYP	Rotterdam criteria	3	T; LH; FSH
	CG	50	28.25 ± 6.71	Diane-35		3	
Jiang (2022) (25)	EG	60	30.88 ± 4.32	LE + ZYP	Rotterdam criteria	3	LH, FSH and E_2_ of hCG trigger day; Endometrial thickness; Pregnancy rate; Ovulation rate; Miscarriage rate
	CG	60	29.75 ± 6.32	LE		3	
Tan (2021) (36)	EG	56	NM	CC + ZYP	Rotterdam criteria	1-6	FSH; LH; Pregnancy rate
	CG	56	NM	CC		1–6	
Li Y (2019) (32)	EG	30	NM	Diane-35 + ZYP	Rotterdam criteria	3	Ovulation rate; T; LH; FSH
	CG	30	NM	Diane-35		3	
Lin (2022) (33)	EG	74	29.45 ± 2.19	CC + ZYP	Rotterdam criteria	1–6	T; LH; Endometrial thickness; Pregnancy rate
	CG	74	29.36 ± 2.11	CC		1–6	
Luo (2022) (34)	EG	50	28.11 ± 2.01	CC + ZYP	Rotterdam criteria	3	Pregnancy rate; Ovulation rate; Miscarriage rate
	CG	50	27.88 ± 2.03	CC		3	
Li (2021 (27)	EG	52	31.2 ± 9.1	LE + ZYP	2006 China PCOS Symposium Expert Consensus	1	Pregnancy rate; Ovulation rate; Endometrial thickness; E_2_
	CG	44	31.2 ± 8.7	LE		1	
Li (2022) (22)	EG	30	28.89 ± 3.01	Stage 1:DET(II)+ZYP Stage 2: LE + DDG + ZYP	Rotterdam criteria	3	Endometrial thickness; E_2_; LH
	CG	30	29.33 ± 3.58	Stage 1:DET(II) Stage 2: LE + DDG		3	
Li (2023) (28)	EG	60	27.14 ± 2.17	Yasmin + ZYP	2018 Chinese guideline	3	FSH; LH; E_2_; T
	CG	60	27.65 ± 2.09	Yasmin		3	
Ren (2022) (35)	EG	43	28.74 ± 2.49	Diane-35 + Met + ZYP	2018 Chinese guideline	3	Pregnancy rate; LH; E_2_; FSH
	CG	43	28.69 ± 2.45	Diane-35 + Met		3	
Li (2024) (29)	EG	40	31.25 ± 5.36	Yasmin + ZYP	2018 Chinese guideline	3	Pregnancy rate; Miscarriage rate; LH; E_2_; FSH; Endometrial thickness
	CG	40	30.74 ± 5.01	Yasmin		3	
Xu (2021) (39)	EG	30	27.43 ± 3.89	Stage 1:Diane-35 + ZYP Stage 2:LE + CGI (administer urofollitropin when necessary)	2018 Chinese guideline	3	Pregnancy rate; Miscarriage rate; LH; T
	CG	30	27.07 ± 3.49	Stage 1:Diane-35 Stage 2: LE + CGI (administer urofollitropin when necessary)		3	
Xu (2008) (38)	EG	48	27.6	CC + ZYP	unspecified	3	Pregnancy rate; Ovulation rate; Miscarriage rate
	CG	48		CC		3	
Li (2017) (30)	EG	45	29.2	Diane-35 + ZYP	Rotterdam criteria	3	LH; FSH; E_2_
	CG	45	30.1	Diane-35		3	
Li F (2024) (26)	EG	45	30.43 ± 3.34	CGI + ZYP	2018 Chinese guideline	3	Pregnancy rate; Ovulation rate;LH; FSH; E_2_; Endometrial thickness
	CG	45	30.22 ± 3.20	CGI		3	
Wang (2024) (37)	EG	51	30.67 ± 4.32	LE + DDG + CGI + ZYP	2018 Chinese guideline	3	Pregnancy rate; Ovulation rate; Miscarriage rate; LH; FSH; E_2_; Endometrial thickness
	CG	51	31.43 ± 4.81	LE + DDG + CGI		3	

EG, experimental group; CG, control group; NM, no mention; LE, letrozole; DDG, dydrogesterone; ZYP, Zishen Yutai Pill; CC, clomiphene citrate; DET (II), drospirenone and ethinylestradiol tablets (Ⅱ); Met, metformin; CGI, chorionic gonadotropin for injection; hCG, human Chorionic gonadotropin.

2018 Chinese guideline: Chinese guideline for diagnosis and management of polycystic ovary syndrome*.*

Rotterdam criteria: revised 2003 consensus on diagnostic criteria and long-term health risks related to polycystic ovary syndrome*.*

2006 China PCOS symposium expert consensus; minutes of the 2nd Chinese national symposium on recent advances in diagnosis and management of polycystic ovary syndrome and related disorders.

In all the studies, ZYP was supplied by the same pharmaceutical company, formulated as pills, with a consistent dosage of 5 g per administration and three administrations daily.

### 3.3 Risk of bias assessment

**Figure 2:**
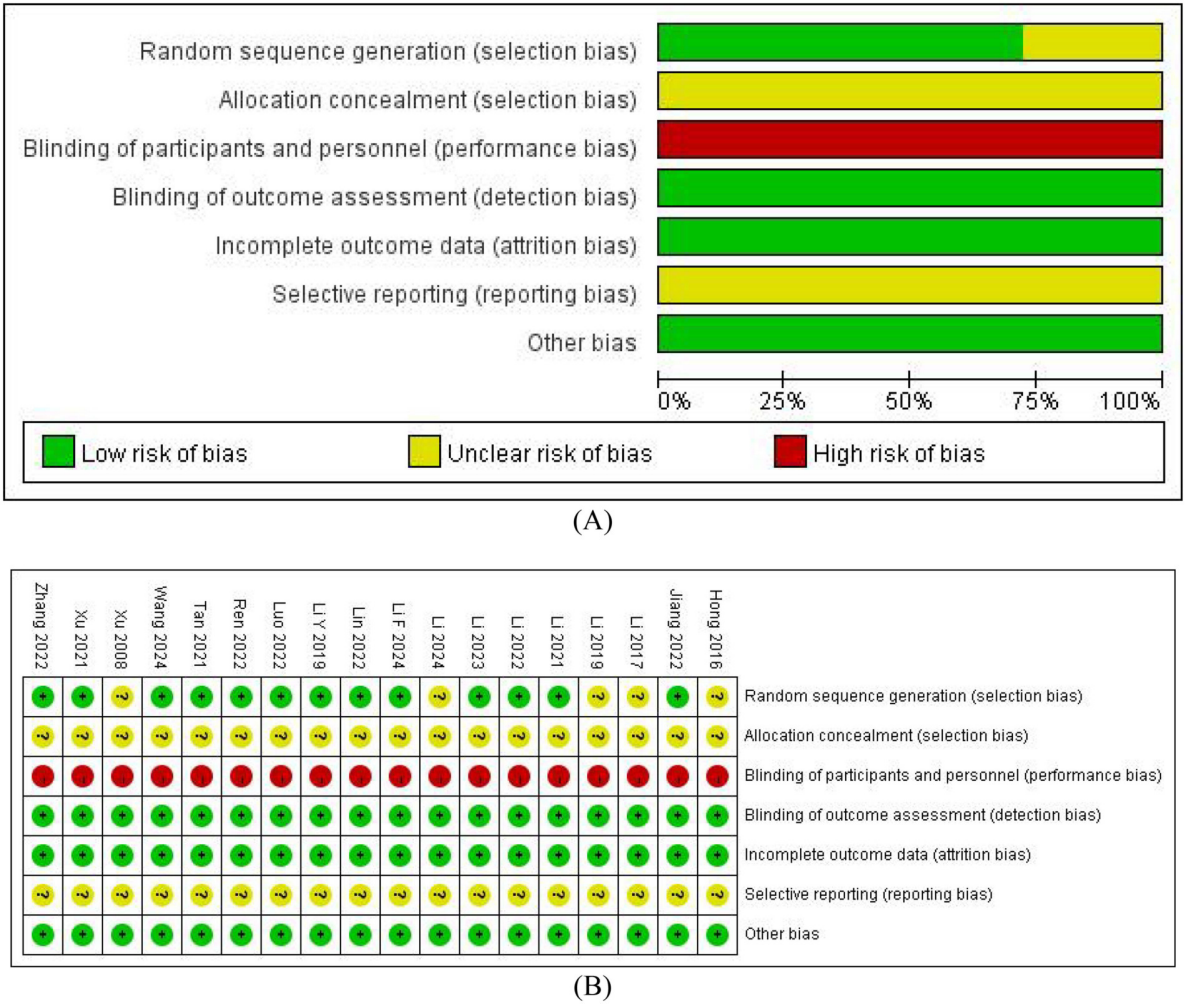
Summary chart of bias risk. **(A)** Each risk of bias item presented as percentages across all included studies. **(B)** Each risk of bias item for each included study.

The risk of bias assessment revealed that most studies had unclear or high risk of bias across multiple domains (Figure 2). Allocation concealment and blinding were not clearly described in most studies, contributing to overall concerns about bias. Randomization methods were adequately described in only a subset of studies. These limitations were considered in the GRADE assessment of evidence quality.

The results of publication bias are presented in the supplementary materials (Supplementary File 1). For outcomes with ≥10 studies (pregnancy rate, LH, FSH), Egger's test found significant publication bias in pregnancy rate. The trim-and-fill adjustment reduced the effect size but retained significance and directionality, confirming the robustness of the result. No bias was found in LH or FSH, and heterogeneity was negligible. For outcomes with fewer studies, a visual assessment of funnel plots suggested potential bias in ovulation rates, although adjusted effects remained significant despite increased heterogeneity. Asymmetry in miscarriage rate and endometrial thickness did not affect effect estimates after trim-and-fill, possibly due to low study power. For T, the observed asymmetry conflicted with trim-and-fill results, and high heterogeneity required caution. E2 showed no bias.

### 3.4 Primary and secondary outcomes

#### 3.4.1 Pregnancy rate

**Figure 3:**
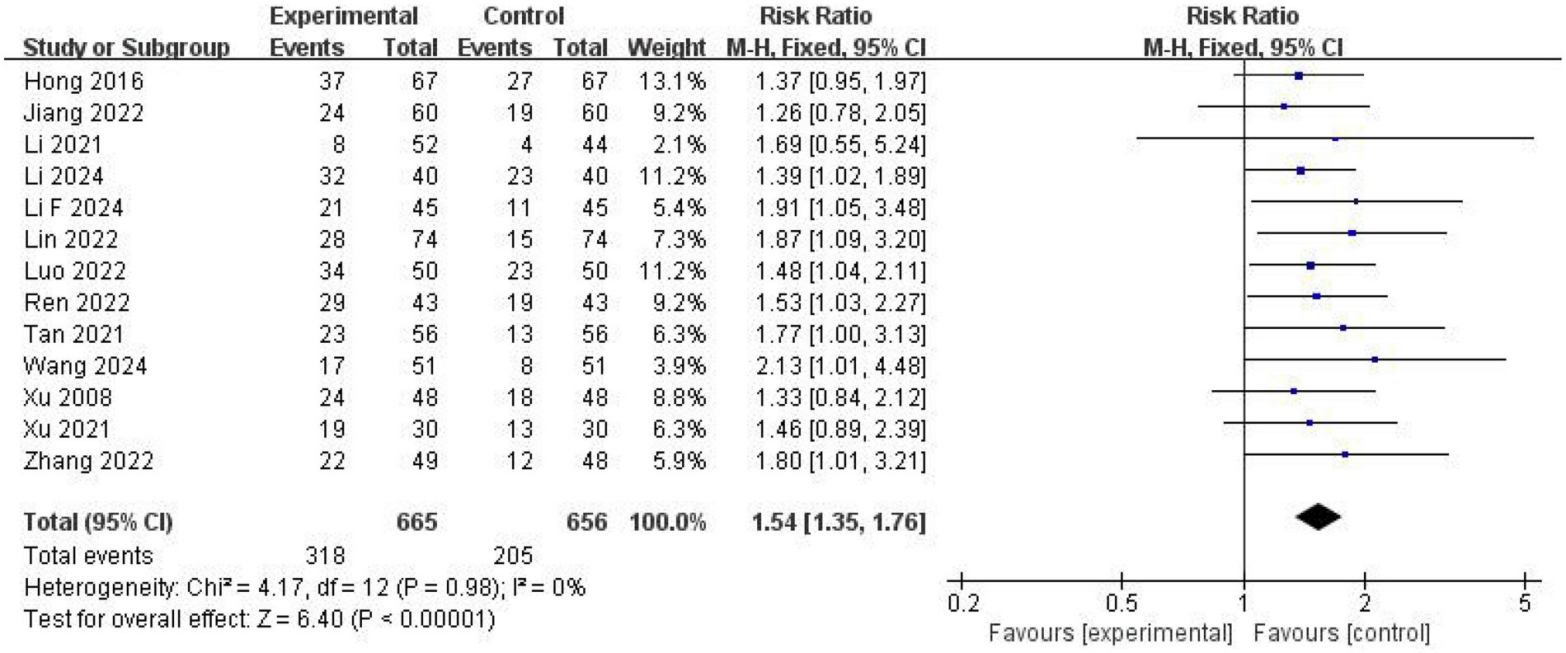
Meta-analysis of pregnancy rate.

Thirteen studies (24–27, 29, 33–40) reported pregnancy rates, encompassing 665 and 656 patients in experimental and control groups respectively (Figure 3). A fixed-effects model was employed for the pooled analysis, as no significant heterogeneity was detected among the studies (I^2^ = 0%, *P* = 0.98). Analysis revealed that pregnancy rates were significantly higher in the experimental group, which received ZYP in addition to Western medicine, compared with the control group that received Western medicine alone (RR = 1.54, 95% CI = 1.35–1.76, *P* < 0.00001), indicating a beneficial effect of ZYP. Although pregnancy definitions varied across studies, statistical heterogeneity was negligible, suggesting that definitional differences were unlikely to be a major source of between-study variability. Sensitivity analyses using sequential exclusion of individual studies demonstrated robust and consistent estimates for pregnancy rates.

#### 3.4.2 Ovulation rate

**Figure 4:**
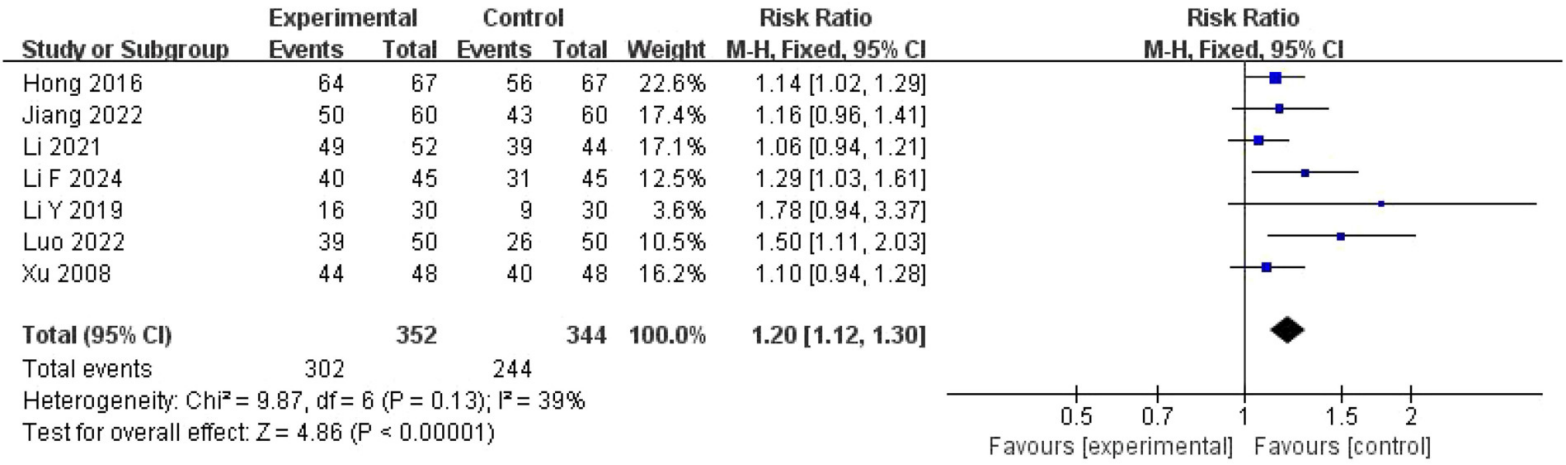
Meta-analysis of ovulation rate.

Data on ovulation rates were obtained from seven RCTs (22, 24–26, 32, 34, 38) with 352 participants in the experimental group and 344 participants in the control group (Figure 4). One study by Wang et al. was excluded from this analysis as it reported cycle ovulation rate (number of ovulatory cycles divided by total cycles observed) rather than patient ovulation rate (number of ovulating patients divided by total patients in each group).

Heterogeneity was not detected (*I*^2^ = 39%, *P* = 0.13); therefore, ovulation rate was analyzed using a fixed-effects model. A significant difference was detected between the experimental and control groups (RR = 1.20, 95% CI = 1.12–1.30, *P* < 0.00001), indicating that ZYP, combined with Western medicines, was superior to Western medicines alone in improving ovulation rates. Sensitivity analysis for ovulation rate revealed no substantial deviation in either the magnitude or direction of the effect size, underscoring the robustness of the study findings.

#### 3.4.3 Miscarriage rate

**Figure 5:**
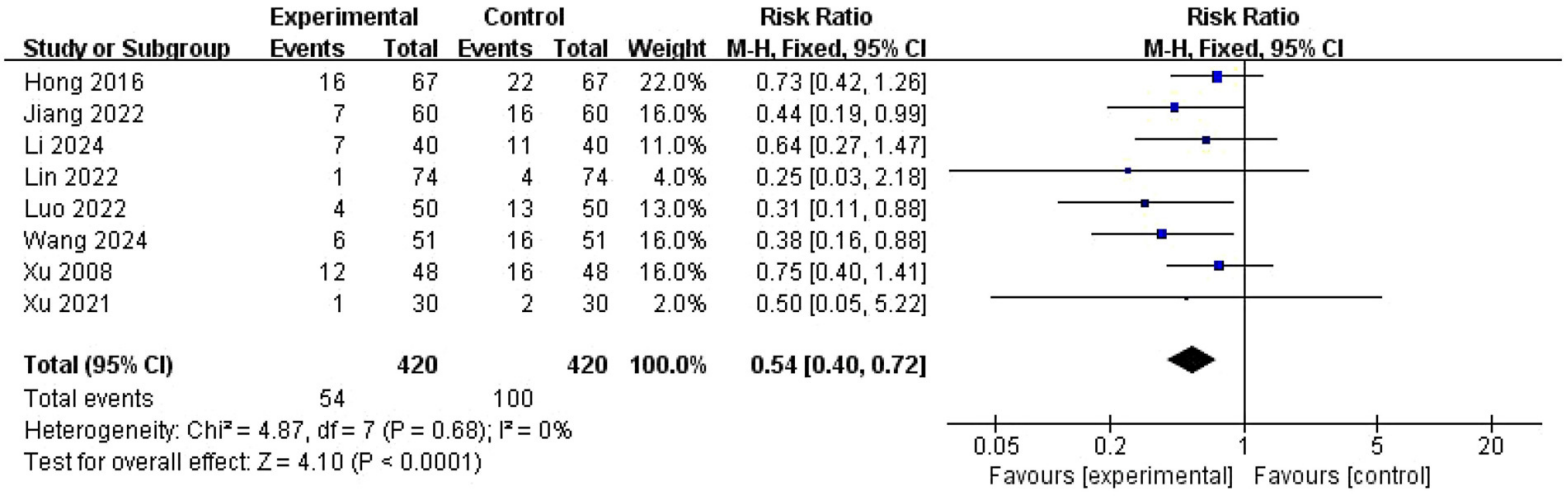
Meta-analysis of miscarriage rate.

Eight studies (24, 25, 29, 33, 34, 37–39) reported miscarriage rates, with 420 participants in each treatment group (Figure 5). Consistency was maintained across studies (*I*^2^ = 0%, *P* = 0.68), and a fixed-effects model was used to compare miscarriage rates between groups. The miscarriage rate was significantly lower in the experimental group compared with the control group (RR = 0.54, 95% CI = 0.40–0.72, *P* < 0.0001), suggesting that ZYP has a beneficial effect on the miscarriage rate in PCOS patients. Sensitivity analysis validated the result stability.

#### 3.4.4 Testosterone (T) levels

**Figure 6:**
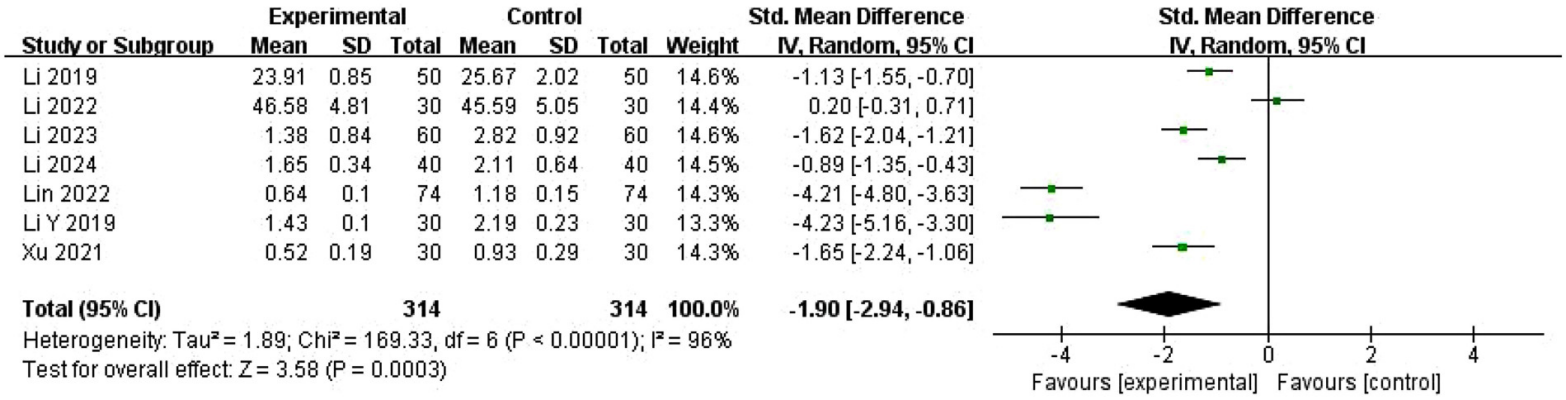
Meta-analysis of the changes in T level.

Data on T levels were analyzed in seven RCTs (22, 28, 29, 31–33, 39) including 628 patients (Figure 6). Given the high heterogeneity observed in these studies (*I*^2^ = 96%, *P* < 0.00001), a random-effects model was used for pooled analysis. Results indicated that ZYP combined with Western drugs significantly reduced T levels compared with Western drugs alone (SMD = −1.90, 95% CI = −2.94 to −0.86, *P* = 0.0003). Although all included studies reported T decreases in both experimental and control groups, six studies (28, 29, 31–33, 39) showed statistically significant lower levels in the experimental group compared with the control group. However, one study (22) reported higher T levels in the experimental group, although the difference was not statistically significant.

To explore the sources of heterogeneity, meta-regression analysis was performed (Supplementary Table 2). Univariate meta-regression identified significant covariates, including CM syndrome differentiation (coefficient = 2.497, 95% CI: 0.827–4.168, *P* = 0.003), treatment duration (coefficient = −2.699, 95% CI: −5.74 to 0.345, *P* = 0.082), region (coefficient = 1.414, 95% CI: 1.048–1.779, *P* = 0.000), and Western medicine type (coefficient = −2.699, 95% CI: −5.740–0.345, *P* = 0.082). Due to the limited number of studies, a multivariate meta-regression was not conducted, and exploratory subgroup analyses were performed instead.

Subgroup results showed that CM syndrome-differentiated ZYP use yielded more pronounced T reduction (SMD = −3.35, 95% CI: −5.17 to −1.54, *P* < 0.00001) than non-differentiated use (SMD = −0.87, 95% CI: −1.59 to −0.15, *P* < 0.00001) (Supplementary Figure 1). For Western medicine types, ovulation induction agents combined with ZYP showed the greatest T-lowering effect (SMD = −4.21, 95% CI: −4.80 to −3.63), followed by menstrual regulators (SMD = −1.49, 95% CI: −2.32 to −0.67) (Supplementary Figure 2). However, high within-subgroup heterogeneity in both syndrome differentiation and Western medicine type analyses suggested these factors poorly explained heterogeneity.

**Figure 7:**
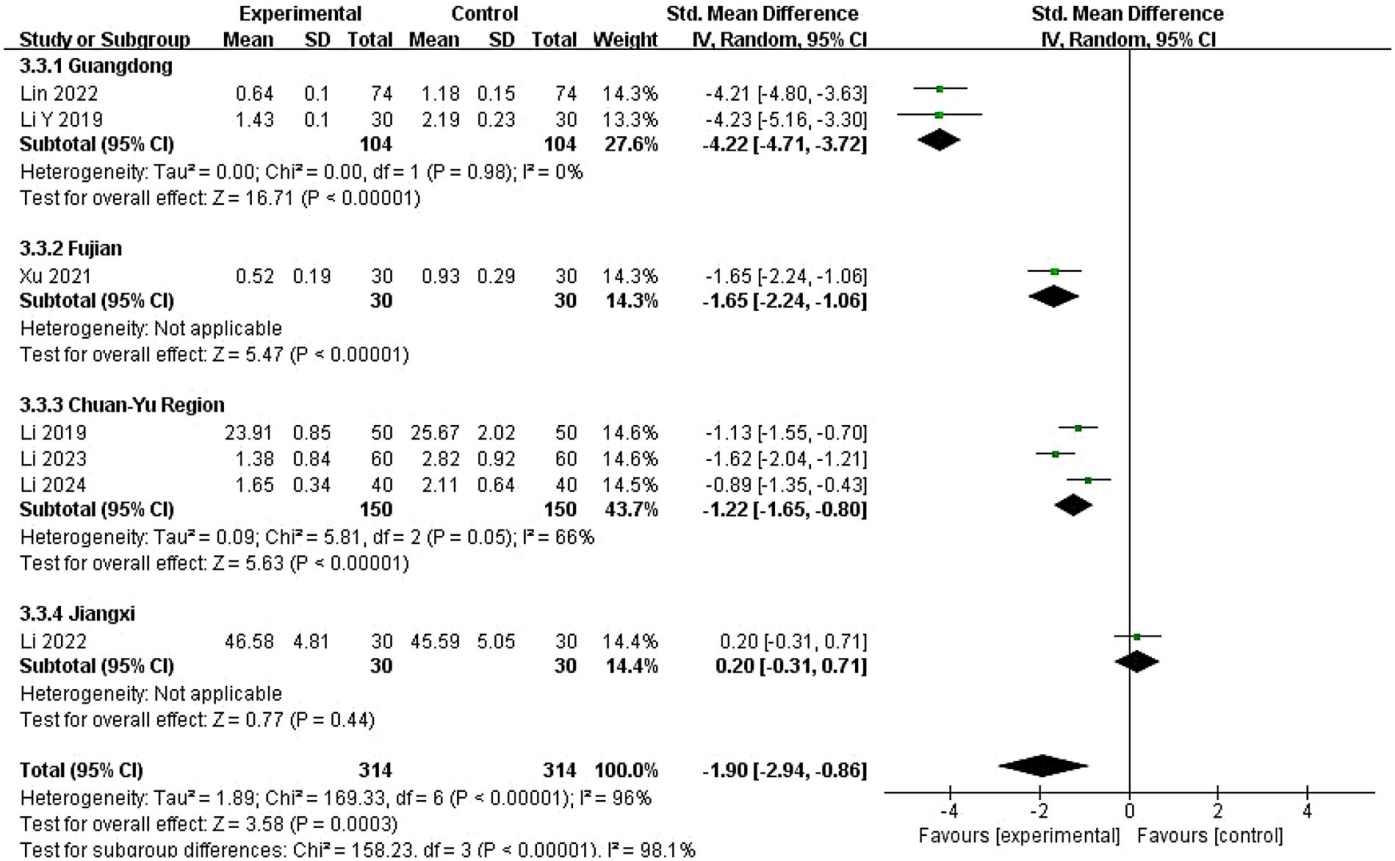
Subgroup analysis by regional distribution of PCOS patients of T.

Regional subgroup analysis revealed the Guangdong group had the largest T reduction (SMD = −4.22, 95% CI: −4.71 to −3.72) with minimal heterogeneity, followed by Fujian (SMD = −1.65, 95% CI: −2.24 to −1.06) and Chuan-Yu (SMD = −1.22, 95% CI: −1.65 to −0.80), the latter showing modest heterogeneity. The Jiangxi subgroup (SMD = 0.20, 95% CI: −0.31 to 0.71) showed no significant reduction in T. Regional subgrouping significantly reduced heterogeneity, indicating that patient geography is a key source of heterogeneity in PCOS studies (Figure 7). Further rigor was confirmed through sensitivity analysis, which supported the robustness of pooled SMD estimates (Supplementary Figure 3).

#### 3.4.5 Luteinizing hormone (LH)

**Figure 8:**
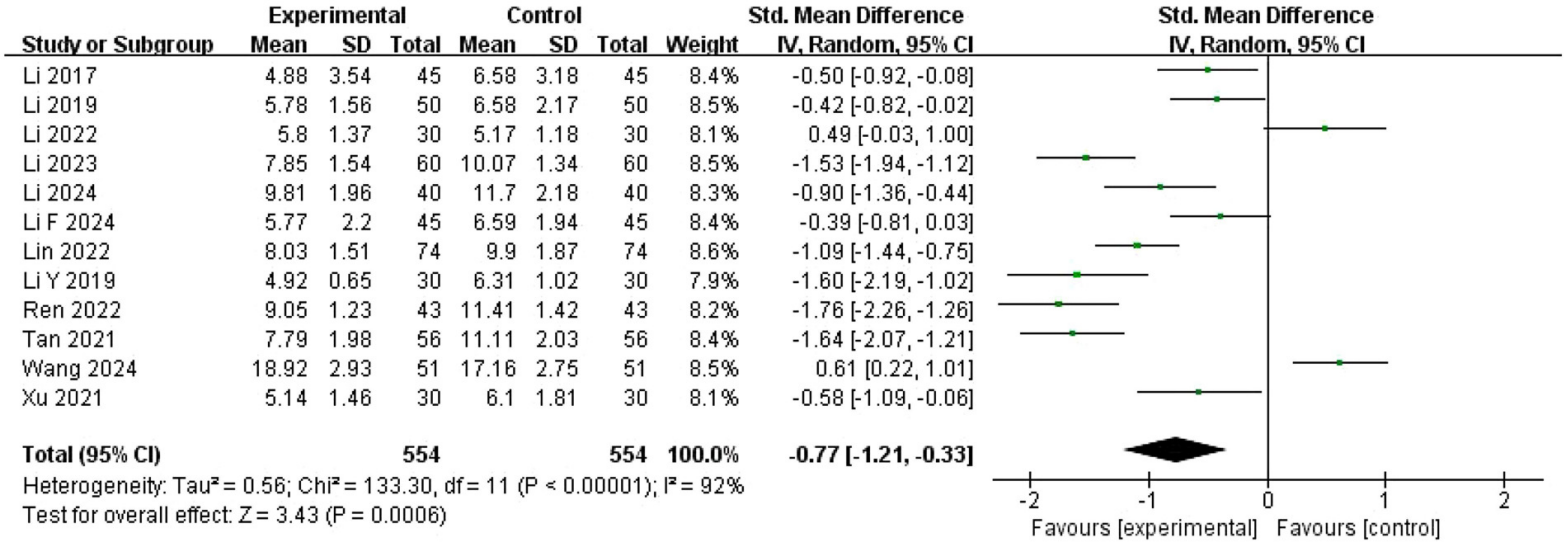
Meta-analysis of the changes in LH level.

Thirteen studies (22, 25, 26, 28–33, 35–37, 39) reported LH levels. However, one study (25) was excluded from meta-analysis because it compared LH levels on the third day of the menstrual cycle and on the day of hCG injection, which did not align with our focus on changes in sex hormone levels during the follicular phase (Figure 8). The remaining 12 studies (22, 26, 28–33, 35–37, 39) exhibited high heterogeneity (*I*^2^ = 92%, *P* < 0.00001); thus, a random-effects model was employed for analysis. Analysis revealed that LH levels were significantly lower in the experimental group compared with the control group (SMD = −0.77, 95% CI = −1.21 to −0.33, *P* = 0.0006). Each of these 12 studies reported significant reductions in LH levels in both treatment groups after treatment.

Univariate meta-regression (Supplementary Table 3) identified PCOS comorbid infertility status as a significant predictor (coefficient = −1.145, 95% CI: −2.003 to −0.287, *P* = 0.009). Although Western medicine type showed no statistical significance (coefficient = −0.273, 95% CI: −0.994 to 0.448, *P* = 0.458), it was considered clinically relevant to LH reduction by ZYP. After excluding multicollinearity via VIF calculation (VIF = 1.00), both Western medicine type and infertility status were included in multivariate meta-regression.

The multivariate model confirmed infertility status as a significant predictor of effect size, with a negative coefficient indicating an inverse association (coefficient = −1.110, 95% CI: −2.009 to −0.211, *P* = 0.016) (Supplementary Table 4). This suggests ZYP may have a weaker LH-lowering effect in PCOS patients with comorbid infertility compared with those without. Consecutive subgroup analysis supported this: non-infertile patients showed more pronounced intervention effect (SMD = −1.63, 95% CI: −1.91 to −1.35), while infertile patients had weaker effect (SMD = −0.50, 95% CI: −0.97 to −0.03). Notably, the infertile subgroup exhibited extreme heterogeneity (I^2^ = 90.9%), whereas the non-infertile subgroup showed no heterogeneity (I^2^ = 0.0%), indicating overall heterogeneity originated primarily from infertile patients (Supplementary Figure 4). Sensitivity analyses confirmed the robustness of meta-analysis results for LH (Supplementary Figure 5).

#### 3.4.6 Follicle-stimulating hormone (FSH)

**Figure 9:**
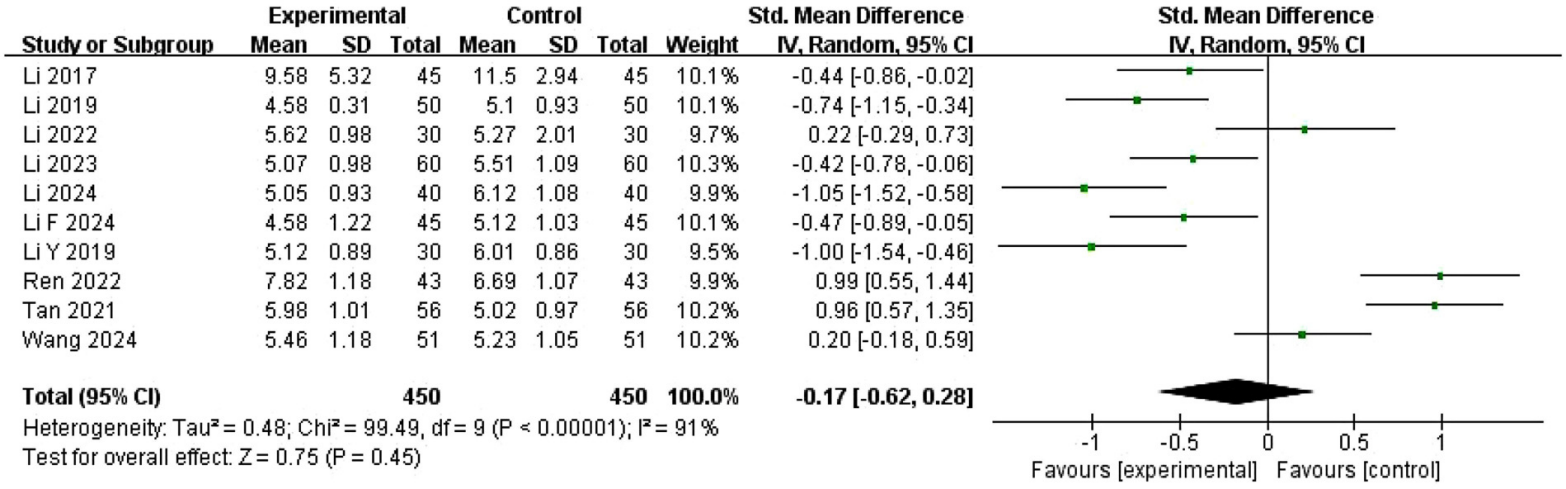
Meta-analysis of the changes in FSH level.

Ten studies (22, 26, 28–32, 35–37) with 450 cases reported serum FSH levels measured on days 2-5 of the menstrual cycle before and after treatment (Figure 9). Heterogeneity among these studies was high (*I*^2^ = 91%, *P* < 0.00001), and treatment groups were therefore compared using a random-effects model. Pooled results showed that FSH in the experimental group was lower than in the control group after treatment, but the difference was not significant (SMD = −0.17, 95% CI = −0.62 to 0.28, *P* = 0.45).

Four of these ten studies (22, 35–37) showed significantly higher FSH in the experimental group compared with the control group, while results were reversed in the remaining six studies (26, 28–32). Seven studies (26, 28–32, 37) showed a decrease in FSH in both experimental and control groups after treatment, whereas in studies conducted by Tan et al. (36) and Ren et al. (35), FSH levels in both groups were elevated after treatment. The study of Li et al. (22) reported FSH decrease in the experimental group and an increase in the control group after treatment, with higher FSH levels in the experimental group compared with the control group, but none of these results were statistically significant.

Univariate meta-regression identified five significant covariates (*P* < 0.1), including region (coefficient = 0.314, 95% CI: 0.102–0.525, *P* = 0.004), Western medicine type (coefficient = 0.799, 95% CI: 0.322–1.276, *P* = 0.001), ZYP administration method (coefficient = 0.367, 95% CI: −0.037 to 0.770, *P* = 0.075), treatment duration (coefficient = 1.272, 95% CI: −0.080 to 2.624, *P* = 0.065), and randomization flaws (coefficient = −0.826, 95% CI: −1.727 to 0.075, *P* = 0.072) (Supplementary Table 5). Western medicine type and ZYP administration method (VIF = 1.00) were selected for multivariate meta-regression. The multivariate model indicated that Western medicine type (coefficient = 0.69, 95% CI: 0.22–1.16, *P* = 0.004) was a potential source of heterogeneity, while the ZYP administration method showed no significant effect on the effect size (coefficient = 0.23, 95% CI: −0.07–0.53, *P* = 0.137) (Supplementary Table 6). Subgroup analysis by Western medicine type was performed for validation, but moderate-to-high within-subgroup heterogeneity precluded identification of heterogeneity sources (Supplementary Figure 6). Sensitivity analysis found that deleting any study did not affect the overall combined estimate, indicating result's rigor (Supplementary Figure 7).

#### 3.4.7 Estradiol (E2)

**Figure 10:**
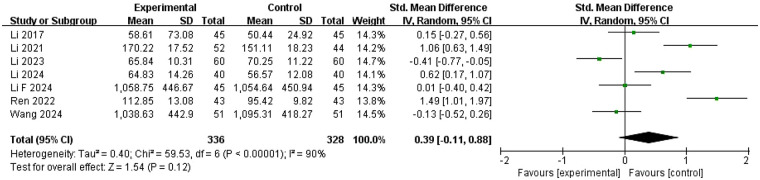
Meta-analysis of the changes in E2 level.

Changes in E2 levels during the follicular phase were reported in seven studies (22, 26–28, 30, 35, 37) involving 664 women (Figure 10). Given significant heterogeneity observed across these studies (*I*^2^ = 90%, *P* < 0.00001), a random-effects model was used for analysis. Results indicated that the difference in E2 levels between experimental and control groups was not statistically significant (SMD = 0.39, 95% CI = −0.11 to 0.88, *P* = 0.11).

Two studies (28, 30) reported decreased E2 levels in both experimental and control groups post-treatment, with lower levels observed in the experimental group. Conversely, the other five studies (22, 26, 27, 35, 37) reported an increase in E2 post-treatment, with significantly higher levels in the experimental group compared with the control group.

To explore heterogeneity sources, univariate meta-regression identified one significant covariate: infertility duration (coefficient = −0.625, 95% CI: −1.007 to −0.244, *P* = 0.001) (Supplementary Table 7). Studies were stratified into three subgroups by duration: 3–4 years, ≥4 years, and unknown duration. Subgroup analysis showed that ZYP intervention significantly increased E2 levels in the 3–4 years group (SMD = 1.06, 95% CI: 0.58–1.55, *P* = 0.031), while no significant changes were observed in the ≥4 years group (SMD = −0.07, 95% CI: −0.35 to 0.22, *P* = 0.627) or the unknown duration group (SMD = −0.14, 95% CI: −0.69 to 0.41, *P* = 0.046). However, high within-subgroup heterogeneity persisted, leaving the underlying sources of heterogeneity unresolved (Supplementary Figure 8). Sensitivity analyses confirmed result stability and reliability (Supplementary Figure 9).

#### 3.4.8 Endometrial thickness

**Figure 11:**
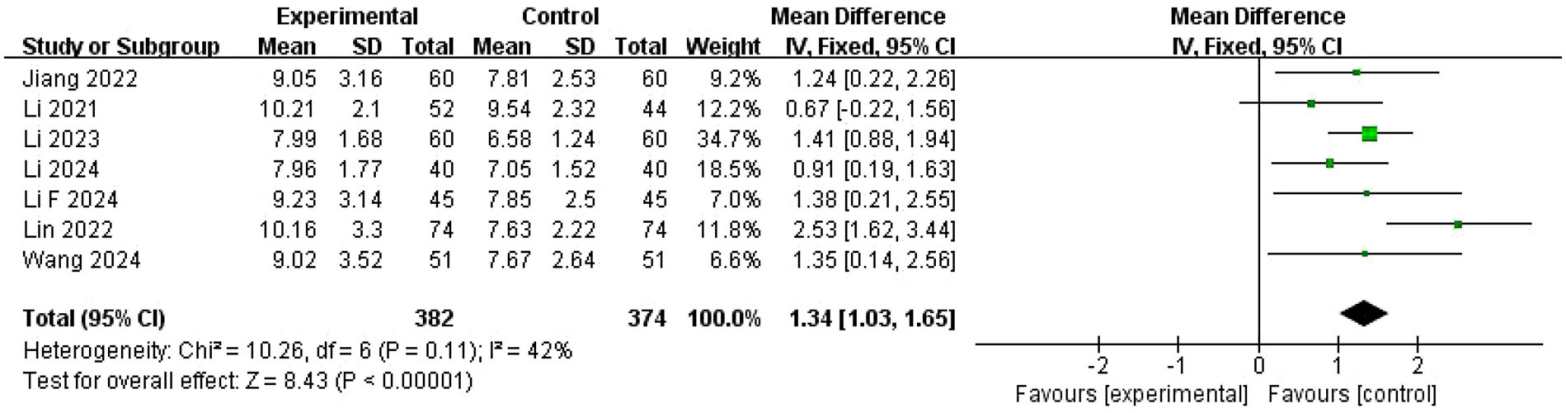
Meta-analysis of the changes in endometrial thickness

Seven studies (25–29, 33, 37) including 756 patients documented changes in endometrial thickness (Figure 11). Significant heterogeneity was found among these studies (*I*^2^ = 42%, *P* = 0.11), and treatment groups were therefore compared using a fixed-effects model. Pooled analysis showed that endometrial thickness was significantly increased in the experimental group compared with the control group (SMD = 1.34, 95% CI = 1.03–1.65, *P* < 0.00001). Sensitivity analyses confirmed result rigor (Supplementary Figure 10.).

### 3.5 Quality of evidence

A systematic review of RCTs testing the efficacy of ZYP combined with Western medications in treating polycystic ovary syndrome (PCOS) was conducted. Eighteen studies of moderate to extremely low quality (22, 24–40) were identified. All included studies had a risk of bias, as allocation concealment and blinding were not clearly described, which downgraded the evidence for all outcomes by one level. Evidence regarding ZYP effects on FSH, T, LH, and E2 was downgraded by two levels due to substantial heterogeneity among studies on these endpoints.

**Figure 12:**
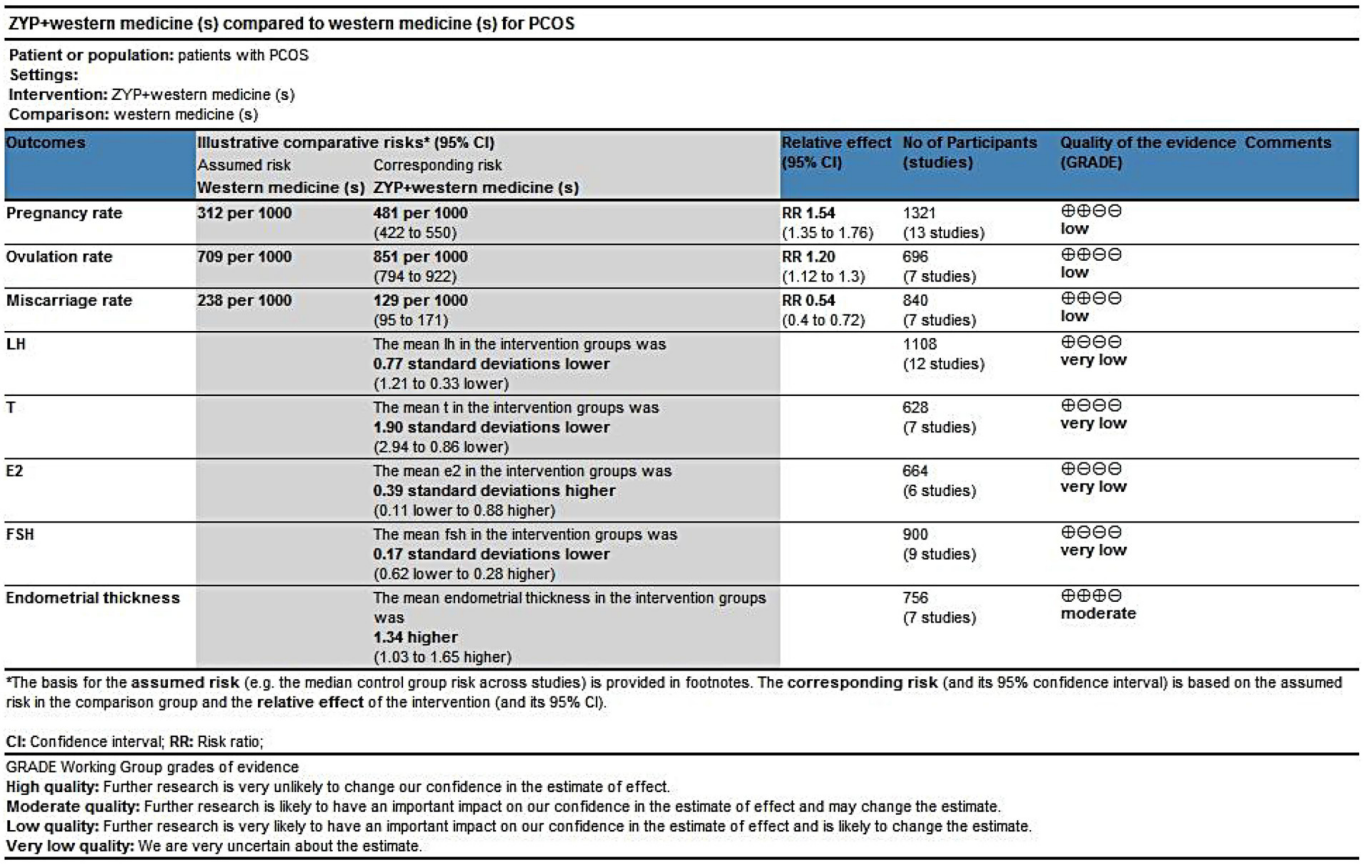
GRADE evidence profile of the results in this systematic review.

Regarding publication bias, after evaluating Egger's test results and funnel plots, evidence levels for pregnancy rate, miscarriage rate, endometrial thickness, T, and ovulation rate were downgraded. The pooled effect size for endometrial thickness was large, so the evidence level for this outcome was upgraded by one level. In summary, the evidence quality for endometrial thickness was moderate, while for pregnancy rate, ovulation rate, and miscarriage rate, it was low; and for T, LH, FSH, and E2, it was extremely low. Results are presented in Figure 12.

ZYP may improve selected reproductive outcomes, while evidence for hormonal effects remains very uncertain. Interpretation should be cautious due to low or very low certainty of evidence and prevalent methodological limitations. Differences in pregnancy outcome definitions across trials were considered when rating the certainty of evidence.

### 3.6 Heterogeneity analysis and meta-regression results

#### 3.6.1 Overview of heterogeneity sources

The meta-regression and subgroup analyses revealed several clinically relevant patterns in treatment response heterogeneity across the primary and secondary outcomes. Significant heterogeneity was observed for testosterone (*I*^2^ = 96%), LH (*I*^2^ = 92%), FSH (*I*^2^ = 91%), and E2 (*I*^2^ = 90%) outcomes, necessitating a comprehensive exploration of potential sources through univariate and multivariate meta-regression analyses. The heterogeneity investigation identified multiple patient and treatment-related factors that significantly influenced ZYP treatment response, providing valuable insights for clinical practice optimization.

#### 3.6.2 Testosterone reduction heterogeneity

For testosterone reduction, three key factors emerged with important clinical implications. Geographic region demonstrated the strongest influence on treatment response, with patients from Guangdong province showing significantly stronger responses than other regions (SMD = −4.22, 95% CI: −4.71 to −3.72), suggesting potential genetic or environmental influences on ZYP efficacy. Regional subgrouping significantly reduced heterogeneity, indicating that patient geography is a key source of heterogeneity in PCOS studies.

CM syndrome differentiation-guided ZYP application significantly enhanced testosterone-lowering effects, with differentiated use yielding more pronounced T reduction (SMD = −3.35, 95% CI: −5.17 to −1.54, *P* < 0.00001) compared to non-differentiated use (SMD = −0.87, 95% CI: −1.59 to −0.15, *P* < 0.00001). This finding highlights the value of personalized CM diagnostic approaches in clinical practice.

Additionally, ZYP combined with ovulation-inducing agents demonstrated superior testosterone reduction (SMD = −4.21, 95% CI: −4.80 to −3.63) compared to combinations with menstrual-regulating drugs (SMD = −1.49, 95% CI: −2.32 to −0.67), suggesting this specific combination may be preferred when hyperandrogenism is a primary concern.

#### 3.6.3 LH reduction heterogeneity

For LH reduction, infertility status emerged as the most significant predictor of treatment response. Multivariate meta-regression confirmed infertility status as a significant predictor of effect size, with a negative coefficient indicating an inverse association (coefficient = −1.110, 95% CI: −2.009 to −0.211, *P* = 0.016). Non-infertile PCOS patients showed markedly stronger LH-lowering effect (SMD = −1.63, 95% CI: −1.91 to −1.35) compared to infertile patients (SMD = −0.50, 95% CI: −0.97 to −0.03), with minimal heterogeneity in the non-infertile group (*I*^2^ = 0.0%) vs. extreme heterogeneity in the infertile subgroup (*I*^2^ = 90.9%). This suggests ZYP may be particularly effective for early PCOS intervention before infertility develops, potentially by addressing hypothalamic-pituitary-ovarian axis dysregulation before it progresses to more resistant states.

#### 3.6.4 FSH and E2 heterogeneity

For FSH and E2 outcomes, Western medication type and infertility duration, respectively, influenced treatment effects, though with persistent heterogeneity. Meta-regression analysis identified Western medicine type as a significant source of heterogeneity for FSH levels (coefficient = 0.69, 95% CI: 0.22–1.16, *P* = 0.004), though the specific effects of different medication combinations require further investigation to fully elucidate the underlying mechanisms.

For E2 levels, univariate meta-regression identified infertility duration as a significant covariate (coefficient = −0.625, 95% CI: −1.007 to −0.244, *P* = 0.001). Subgroup analysis demonstrated that ZYP intervention significantly increased E2 levels in patients with shorter infertility durations of 3-4 years (SMD = 1.06, 95% CI: 0.58–1.55, *P* = 0.031), while no significant changes were observed in patients with ≥4 years duration (SMD = −0.07, 95% CI: −0.35 to 0.22, *P* = 0.627) or unknown duration (SMD = −0.14, 95% CI: −0.69 to 0.41, *P* = 0.046). This reinforces the potential benefits of earlier intervention.

#### 3.6.5 Clinical implications of heterogeneity findings

These heterogeneity findings offer preliminary guidance for clinical decision-making and treatment optimization. First, consider ZYP earlier in the PCOS disease course, particularly before infertility develops, as evidenced by the superior LH-lowering effects in non-infertile patients and enhanced E2 modulation in patients with shorter infertility durations. Second, when possible, incorporate CM syndrome differentiation to optimize treatment response, as demonstrated by the significantly enhanced testosterone-lowering effects with personalized diagnostic approaches. Third, for patients with significant hyperandrogenism, consider combining ZYP with ovulation-inducing agents rather than menstrual regulators, given the superior testosterone reduction observed with this combination. Fourth, recognize that treatment response may vary by geographic region, potentially reflecting genetic or environmental factors that warrant further investigation in future studies.

## 4 Discussion

This meta-analysis, incorporating 18 RCTs (22, 24–40) with 1,751 participants, comprehensively evaluated ZYP's therapeutic effect in combination with Western medicines for treating PCOS. The analysis found that combined treatment significantly improved several clinical outcomes related to PCOS, including increased ovulation rate, pregnancy rate, and endometrial thickness, as well as decreased levels of T and LH. However, no significant effects were observed on E2 and FSH.

Hyperandrogenism (HA), prevalent in 60%–80% of PCOS patients (41), contributes to follicular abnormalities and is thought to be linked to upstream dysregulation in the ovarian central nervous system. This includes abnormal activation of hypothalamic gonadotropin-releasing hormone (GnRH) neurons (42), which increases LH pulse frequency, thereby promoting androgen secretion from ovarian follicular theca cells (43). The elevated androgen levels observed in PCOS patients can disrupt normal follicular development and ovulation (44, 45). This meta-analysis demonstrated that ZYP, when combined with Western medicines, significantly reduced T levels compared with Western medicines alone, suggesting that ZYP may help restore hormonal balance in patients with PCOS.

The reduction in LH levels observed in this analysis aligns with the pathophysiology of PCOS. Elevated LH levels in PCOS patients contribute to increased androgen production and disrupted follicular development (46). By reducing LH levels, ZYP may help normalize the hormonal environment necessary for proper ovulation and follicular maturation. This hormonal improvement likely contributes to the observed increases in ovulation and pregnancy rates.

Estrogen plays crucial roles in follicular development and endometrial preparation for implantation (47). While this analysis did not show significant changes in E2 levels, this may reflect the complex and variable nature of estrogen regulation in PCOS patients. The heterogeneity observed in E2 responses across studies suggests that individual patient characteristics, such as infertility duration, may influence treatment response (48).

The significant improvement in endometrial thickness observed with ZYP treatment is particularly noteworthy. Endometrial receptivity is often compromised in PCOS patients due to hormonal imbalances and oxidative stress (49, 50). Adequate endometrial thickness is crucial for successful implantation and maintaining pregnancy (51). The improvement in endometrial thickness with ZYP treatment may contribute to the observed increases in pregnancy rates and decreases in miscarriage rates.

The mechanisms underlying ZYP's beneficial effects in PCOS may involve multiple pathways. Chinese medicine approaches PCOS from a holistic perspective, addressing underlying constitutional imbalances that contribute to the syndrome (52). ZYP contains multiple herbal components that may work synergistically to regulate hormonal balance, improve ovarian function, and enhance endometrial receptivity (53, 54). Experimental studies have shown that ZYP can modulate various signalling pathways involved in reproductive function, including those affecting hormone synthesis, follicular development, and endometrial angiogenesis (55–57). ZYP contains core active components such as quercetin and isorhamnetin, which can act on targets including CYP19A1 and PI3 K to promote ovarian steroid hormone synthesis and inhibit granulosa cell apoptosis, thereby reducing T levels, regulating the LH/FSH ratio, and providing a favorable hormonal milieu for follicular development (58, 59). In terms of improving pregnancy outcomes, ZYP can significantly promote angiogenesis at the maternal-fetal interface by regulating the miR-187/VEGF axis, enhance embryonic blood supply, boost trophoblast cell viability, inhibit cell apoptosis, and thereby increase the embryo survival rate (57).

The clinical evidence supporting ZYP's efficacy extends beyond the treatment of PCOS. Recent randomized controlled trials have demonstrated ZYP's benefits in other reproductive contexts, including improving live birth rates in fresh embryo transfer cycles (60). This broader evidence base supports the potential therapeutic value of ZYP in reproductive medicine.

These findings should be interpreted in the context of several limitations. First, the quality of evidence was generally low to moderate due to methodological limitations in the included studies, particularly regarding allocation concealment and blinding. Second, significant heterogeneity was observed for several outcomes, which may reflect differences in patient populations, treatment protocols, and outcome measurement methods across studies. Third, most included studies were conducted in Chinese populations, which may limit the generalizability of findings to other ethnic groups. Fourth, BMI, an important modifier of hormonal response and fertility outcomes in PCOS—was inadequately reported in most included trials and therefore could not be analysed. As a result, baseline imbalance and residual confounding related to adiposity cannot be excluded, and the external validity of the findings, particularly for overweight and obese PCOS populations, is limited. Fifth, definitions of pregnancy outcomes were not fully uniform across studies. Variability in pregnancy definitions may introduce outcome misclassification and indirectness, which could affect comparability across studies and the certainty of evidence, even in the absence of statistical heterogeneity. Given that non-ultrasound-based definitions were used in only a small number of trials, formal subgroup analysis was not performed, as such analyses would be underpowered and potentially misleading. Instead, sensitivity analyses we had been performed were considered a more appropriate strategy to evaluate the robustness of the findings.

Despite these limitations, this meta-analysis provides a relatively comprehensive evaluation to date of ZYP's efficacy in the treatment of PCOS. The consistent beneficial effects observed across multiple clinically relevant outcomes support the potential therapeutic value of ZYP as an adjunctive treatment for PCOS. The safety profile of ZYP appears favorable, with no serious adverse events reported in the included studies.

Potential product standardization and sponsorship bias was considered. All included trials used ZYP supplied by a single pharmaceutical manufacturer with identical formulation and dosing. While this product consistency reduces within-study intervention heterogeneity, it may also introduce product-bastardization or sponsorship-related bias, particularly in the context of generally weak trial reporting and limited transparency around randomization and blinding. Even in the absence of declared conflicts of interest, reliance on a single manufacturer may inflate effect estimates and limit external reproducibility if manufacturing processes, quality control, or batch characteristics differ in other settings. These considerations further warrant cautious interpretation of the pooled effects and reinforce the need for independently funded, centimetre trials using rigorously standardised but manufacturer-diverse formulations.

Future research should focus on conducting larger, high-quality randomized controlled trials with standardized protocols and outcome measures. Studies should include diverse patient populations to enhance generalizability and explore optimal dosing regimens and treatment durations. Additionally, mechanistic studies investigating ZYP's molecular targets and pathways of action would provide valuable insights into its therapeutic mechanisms. Importantly, the observed effect sizes—particularly for hormonal endpoints—should be interpreted cautiously, as they are derived predominantly from studies with high or unclear risk of bias and very low GRADE certainty.

## 5 Conclusion

This systematic review and meta-analysis suggests that adjunctive ZYP, when combined with Western medicines, may improve selected reproductive outcomes in women with PCOS, including ovulation rate, pregnancy rate, endometrial thickness, and androgen-related parameters.

However, the certainty of evidence for most outcomes was low or very low, and the majority of included trials were affected by methodological limitations and unclear or high risk of bias. Consequently, these findings should be interpreted as hypothesis-generating rather than practice-changing.

Well-designed, independently funded, multicentre randomised controlled trials with transparent reporting, standardised outcome definitions (including pregnancy outcomes), and adequate adjustment for key confounders such as BMI are required before ZYP can be recommended for routine clinical use or guideline incorporation in PCOS management.

## Data Availability

Publicly available datasets were analyzed in this study. This data can be found here: this study performs a meta-analysis utilizing data extracted from eligible published randomized controlled trials (RCTs).
