# Supplementary material for: Hormonal regulation and reproductive improvement with adjunctive Zishen Yutai Pill in polycystic ovary syndrome: a systematic review with meta-analysis
Source: Front Reprod Health. 2026 Jan 15;7:1748768. doi: 10.3389/frph.2025.1748768 (PMC12852447; doi:10.3389/frph.2025.1748768)
Supplement: Supplementary file 1 [file Table1.docx]

**Supplemental Table**

**Supplemental Table 1. Search strategy in each database**

| **Database** | **Search strategy** |
| --- | --- |
| **PubMed** | (polycystic ovary syndrome OR polycystic ovarian syndrome OR Stein Leventhal Syndrome) AND (randomized controlled trial [pt] OR controlled clinical trial [pt] OR randomized [tiab]) AND (humans [mh] NOT animal [mh]) AND Zishen Yutai Pill |
| **Embase** | #1 ('polycystic ovary syndrome'/de OR 'polycystic ovarian syndrome'/de OR 'Stein Leventhal Syndrome'/de)  #2 ('Zishen Yutai Pill'/de)  #3 ('randomized controlled trial':de OR 'controlled clinical trial':de):de,ab,ti)  #4= #1 AND #2 AND #3 |
| **The Cochrane Library** | #1 polycystic ovary syndrome  #2 Zishen Yutai Pill  #3 = #1 and #2 |
| **Web of Science** | #1 TS= (“polycystic ovary syndrome”) OR (“polycystic ovarian syndrome”) OR (“Stein Leventhal Syndrome”)  #2 TS=(“Zishen Yutai Pill”)  #3 =TS=(“randomized controlled trial”)  #4 = #1 AND #2 AND #3 |
| **ClinicalTrials.gov** | Condition or disease: polycystic ovary syndrome  Intervention/treatment: Zishen Yutai Pill |
| **Chinese National Knowledge**  **Infrastructure (CNKI)** | TI=滋肾育胎丸*(多囊卵巢综合征) AND AB=滋肾育胎丸*(多囊卵巢综合征) |
| **Wanfang** | 主题:(滋肾育胎丸) and 主题:(多囊卵巢综合征)) |
| **VIP Database** | U=滋肾育胎丸 AND (U=多囊卵巢综合征) |
| **Chinese Clinical Trial**  **Registry** | 研究疾病名称：多囊卵巢综合征  干预措施：滋肾育胎丸 |
| **Opengrey** | (polycystic ovary syndrome) and (Zishen Yutai Pill ) |
| **Greyguide** | (polycystic ovary syndrome) and (Zishen Yutai Pill ) |

### Supplemental Table 2. Univariate meta-regression of T.

| **variable** | **Coef.** | **Std. Err.** | **z** | **p_value** | **95% Conf. Interval** | **Number of Study** | **tau^2^ estimate** | **Iteration** |
| --- | --- | --- | --- | --- | --- | --- | --- | --- |
| **var9** | -1.376252 | 1.423675 | -0.97 | 0.334 | [-4.166605, 1.414101] | 7 | 2.781 | 4 |
| **var10** | 2.497064 | 0.852340 | 2.93 | 0.003 | [0.826509, 4.167619] | 7 | 1.147 | 5 |
| **var11** | -0.566752 | 0.628578 | -0.90 | 0.367 | [-1.798742, 0.665237] | 7 | 2.837 | 5 |
| **var12** | -2.699436 | 1.553181 | -1.74 | 0.082 | [-5.743615, 0.344743] | 7 | 1.980 | 7 |
| **var13** | -1.285310 | 1.422306 | -0.90 | 0.366 | [-4.072977, 1.502358] | 7 | 2.824 | 5 |
| **var14** | -0.189611 | 0.611792 | -0.31 | 0.757 | [-1.388702, 1.009480] | 7 | 3.225 | 5 |
| **var15** | -0.855842 | 1.502392 | -0.57 | 0.569 | [-3.800476, 2.088793] | 7 | 3.107 | 5 |
| **var16** | 1.413656 | 0.186550 | 7.58 | <0.001 | [1.048025, 1.779286] | 7 | 0.165 | 4 |
| **var17** | -2.699436 | 1.553181 | -1.74 | 0.082 | [-5.743615, 0.344743] | 7 | 1.980 | 7 |

var9: comorbid infertility status; var10: syndrome-based medication use; var11: infertility duration; var12: treatment course; var13: presence of randomization flaws; var14: ZYP administration method; var15: age; var16: region; var17: Western medicine type.

### Supplemental Table 3. Univariate meta-regression of LH.

| **variable** | **Coef.** | **Std. Err.** | **z** | **p_value** | **95% Conf. Interval** | **No of studies** | **tau² estimate** | **Iteration** |
| --- | --- | --- | --- | --- | --- | --- | --- | --- |
| var9 | -1.144701 | 0.4378628 | -2.61 | 0.009 | [-2.002897, -.02865062] | 12 | 0.3702 | 4 |
| var10 | 0.3849995 | 0.4703751 | 0.82 | 0.413 | [-0.5369188, 1.306918] | 12 | 0.6104 | 4 |
| var11 | -0.1684484 | 0.1888468 | -0.89 | 0.372 | [-0.5385813, 0.2016846] | 12 | 0.6066 | 3 |
| var12 | 0.2304173 | 0.1634401 | 1.41 | 0.159 | [-0.0899194, 0.550754] | 12 | 0.5374 | 4 |
| var13 | -0.7160816 | 0.6034919 | -1.19 | 0.235 | [-1.898904, 0.4667408] | 12 | 0.5647 | 4 |
| var14 | -0.225766 | 0.5547249 | -0.41 | 0.684 | [-1.313007, 0.8614747] | 12 | 0.643 | 4 |
| var15 | 0.3318089 | 0.5036302 | 0.66 | 0.510 | [-0.6552882, 1.318906] | 12 | 0.6211 | 4 |
| var16 | -0.0176297 | 0.5146281 | -0.03 | 0.973 | [-1.026282, 0.9910229] | 12 | 0.6546 | 4 |
| var17 | -0.2729762 | 0.3678997 | -0.74 | 0.458 | [-0.9940463, 0.4480939] | 12 | 0.6187 | 4 |

var9: comorbid infertility status; var10: syndrome-based medication use; var11: infertility duration; var12: ZYP administration method; var13: treatment course; var14: presence of randomization flaws; var15: age; var16: region; var17: Western medicine type.

**Supplemental Table 4. Multivariate meta-regression of LH.**

| **variable** | **Coef.** | **Std. Err.** | **z** | **p_value** | **95% Conf. Interval** | **No of studies** | **tau² estimate** | **Iteration** |
| --- | --- | --- | --- | --- | --- | --- | --- | --- |
| var17 | -.164835 | .3065052 | -0.54 | 0.591 | [-.7655743, .4359042] | 12 | .4021 | 4 |
| var9 | -1.109803 | .4585503 | -2.42 | 0.016 | [-2.008545, -.2110611] | 12 | .4021 | 4 |
| _cons | .8491627 | .7074119 | 1.20 | 0.230 | [-.537339, 2.235664] | 12 | .4021 | 4 |

var9: comorbid infertility status; var17: Western medicine type

### Supplemental Table 5. Univariate meta-regression of FSH.

| **variable** | **Coef.** | **Std. Err.** | **z** | **p_value** | **95% Conf. Interval** | **No of studies** | **tau² estimate** | **Iteration** |
| --- | --- | --- | --- | --- | --- | --- | --- | --- |
| var9 | 0.0507882 | 0.5472551 | 0.09 | 0.926 | [-1.021812, 1.123388] | 10 | 0.577 | 4 |
| var10 | 0.0949919 | 0.5107784 | 0.19 | 0.852 | [-0.9061154, 1.096099] | 10 | 0.5758 | 4 |
| var11 | -0.297698 | 0.2276286 | -1.31 | 0.191 | [-0.7438418, 0.1484458] | 10 | 0.4685 | 3 |
| var12 | 0.3669043 | 0.2058236 | 1.78 | 0.075 | [-0.0365026, 0.7703112] | 10 | 0.3981 | 4 |
| var13 | 1.272369 | 0.6898454 | 1.84 | 0.065 | [-0.0797033, 2.624441] | 10 | 0.3872 | 4 |
| var15 | -0.825751 | 0.459756 | -1.80 | 0.072 | [-1.726856, 0.0753542] | 10 | 0.3949 | 4 |
| var16 | 0.3135185 | 0.1078804 | 2.91 | 0.004 | [0.1020768, 0.5249602] | 10 | 0.2557 | 3 |
| var17 | 0.7991827 | 0.2434919 | 3.28 | 0.001 | [0.3219474, 1.276418] | 10 | 0.216 | 4 |
| var14 | -0.6675593 | 0.4569919 | -1.46 | 0.144 | [-1.563247, 0.2281283] | 10 | 0.45 | 4 |

var9: comorbid infertility status; var10: syndrome-based medication use; var11: infertility duration; var12: ZYP administration method; var13:treatment course; var14: age; var15: presence of randomization flaws; var16: region; var17: Western medicine type.

### Supplemental Table 6. Multivariate meta-regression of FSH.

| **variable** | **Coef.** | **Std. Err.** | **z** | **p_value** | **95% Conf. Interval** | **No of studies** | **tau² estimate** | **Iteration** |
| --- | --- | --- | --- | --- | --- | --- | --- | --- |
| var12 | 0.2291416 | 0.1542601 | 1.49 | 0.137 | [-0.0732026, 0.5314858] | 10 | 0.1823 | 4 |
| var17 | 0.691823 | 0.2388341 | 2.90 | 0.004 | [0.2237168, 1.159929] | 10 | 0.1823 | 4 |
| _cons | -1.581594 | 0.4006496 | -3.95 | 0.000 | [-2.366853, -0.7963352] | 10 | 0.1823 | 4 |

### Supplemental Table 7. Univariate meta-regression of E_2_.

| **variable** | **Coef.** | **Std. Err.** | **z** | **p_value** | **95% Conf. Interval** | **No of studies** | **tau² estimate** | **Iteration** |
| --- | --- | --- | --- | --- | --- | --- | --- | --- |
| var9 | -0.0168705 | 0.2878759 | -0.06 | 0.953 | [-0.5810969, 0.5473559] | 7 | 0.5242 | 4 |
| var10 | 0.5446658 | 0.5256825 | 1.04 | 0.300 | [-0.485653, 1.574985] | 7 | 0.4274 | 4 |
| var11 | 0.440977 | 0.6011725 | 0.73 | 0.463 | [-0.7372995, 1.619253] | 7 | 0.4712 | 3 |
| var12 | -0.6254413 | 0.1944271 | -3.22 | 0.001 | [-1.006512, -0.2443711] | 7 | 0.138 | 4 |
| var13 | 0.3909069 | 0.3727366 | 1.05 | 0.294 | [-0.3396434, 1.121457] | 7 | 0.4248 | 4 |
| var14 | -0.6003486 | 0.458787 | -1.31 | 0.191 | [-1.499555, 0.2988573] | 7 | 0.3773 | 4 |
| var15 | -0.7901727 | 0.7339298 | -1.08 | 0.282 | [-2.228649, 0.6483033] | 7 | 0.414 | 4 |
| var16 | -0.0162508 | 0.2544615 | -0.06 | 0.949 | [-0.5149862, 0.4824847] | 7 | 0.5238 | 4 |
| var17 | -0.0127747 | 0.6327579 | -0.02 | 0.984 | [-1.252957, 1.227408] | 7 | 0.5245 | 4 |
| var18 | 0.010719 | 0.576885 | 0.02 | 0.985 | [-1.119955, 1.141393] | 7 | 0.5245 | 4 |
| var19 | -0.1373788 | 0.2595225 | -0.53 | 0.597 | [-0.6460335, 0.3712759] | 7 | 0.4948 | 4 |

var9: unit of measurement; var10: comorbid infertility status; var11: syndrome-based medication use; var12: infertility duration; var13: Western medicine type; var14: ZYP administration method; var15: treatment course; var16: BMI; var17: presence of randomization flaws; var18: age; var19: region.

**Supplemental Figure**


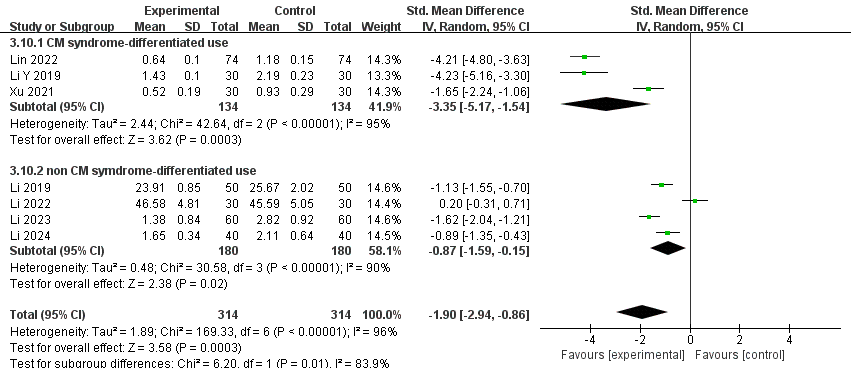


**Supplemental Figure 1. Subgroup analysis by syndrome-based medication use of T.**


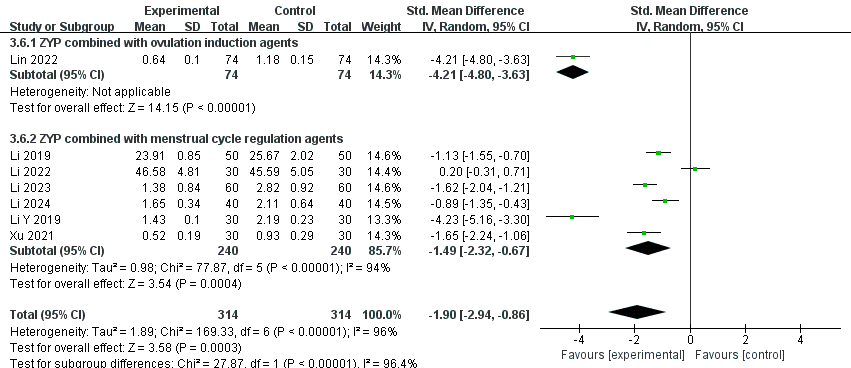


**Supplemental Figure 2. Subgroup analysis by Western medicine types of T.**


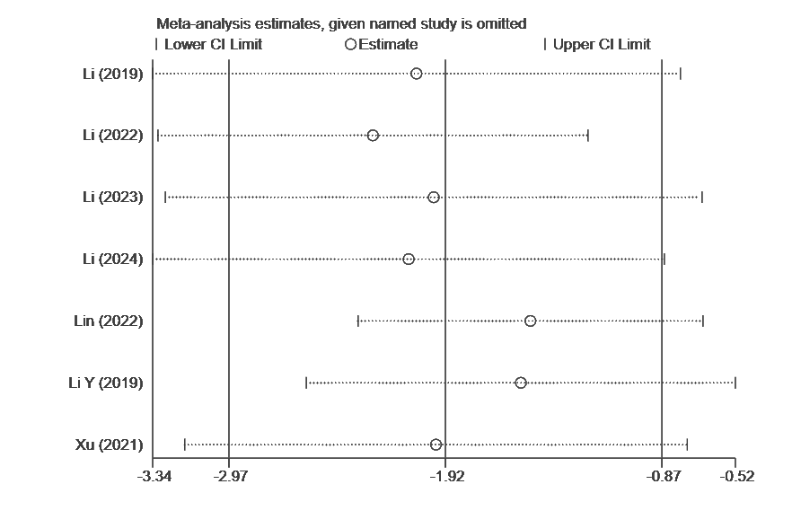


**Supplemental Figure 3. Sensitivity analysis of T.**


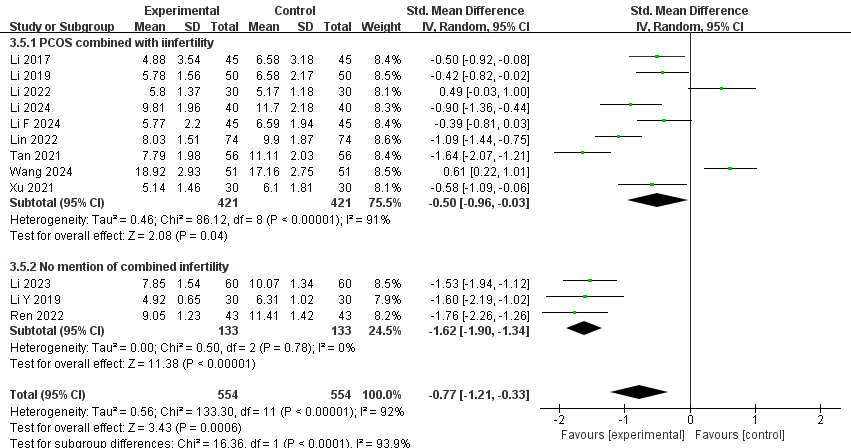


**Supplemental Figure 4. Subgroup analysis by comorbid infertility status of LH.**


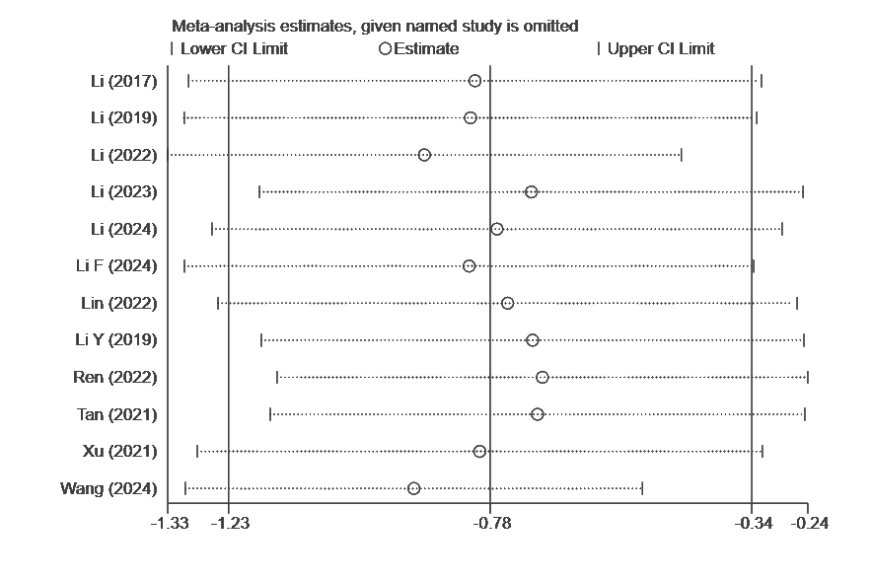


**Supplemental Figure 5. Sensitivity analysis of LH.**


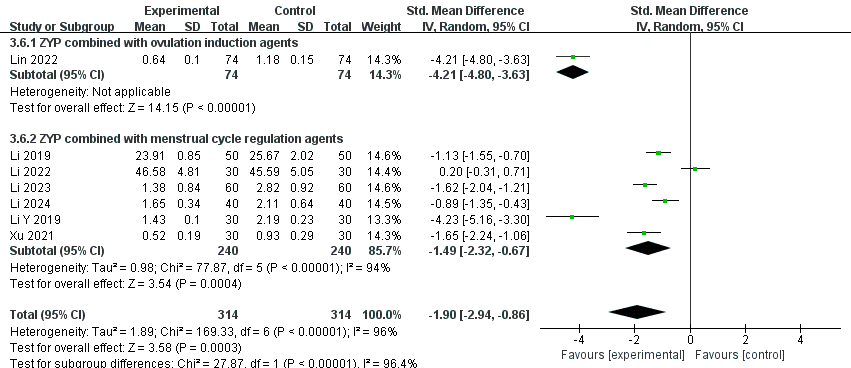


**Supplemental Figure 6. Subgroup analysis by Western medicine types of FSH.**


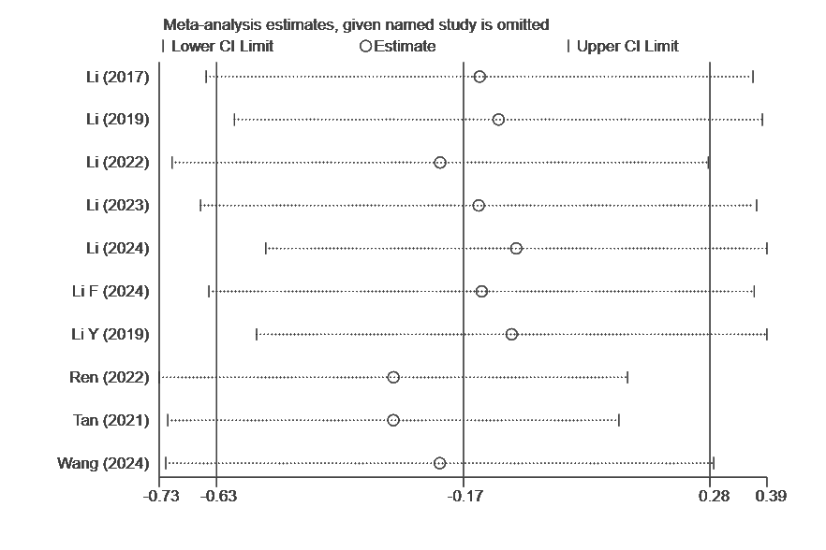


**Supplemental Figure 7. Sensitivity analysis of FSH.**


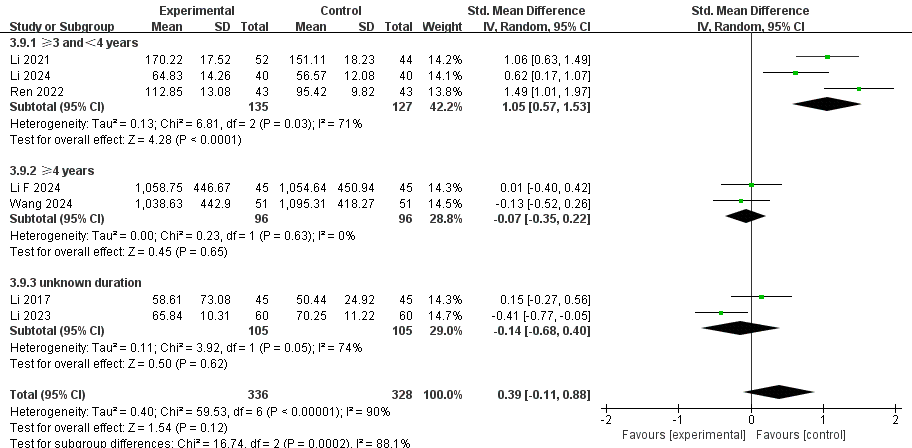


**Supplemental Figure 8. Subgroup analysis by infertility duration of E2.**


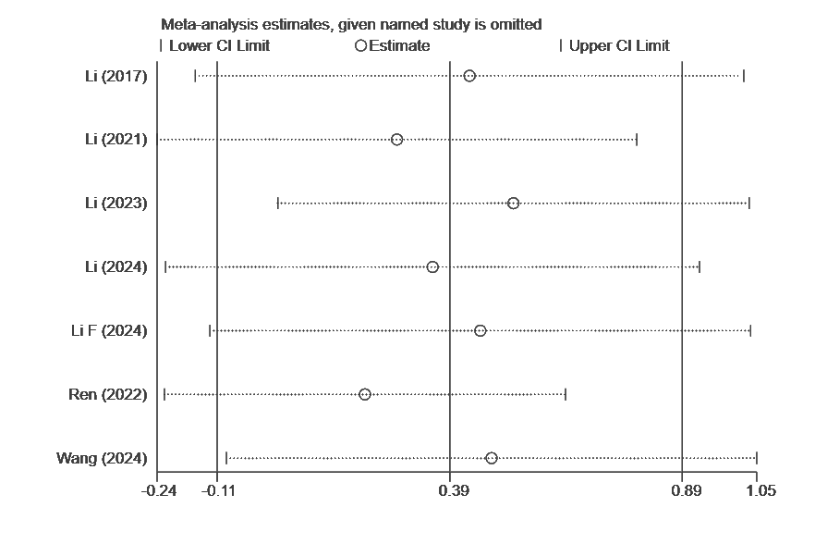


**Supplemental Figure 9. Sensitivity analysis of E2.**


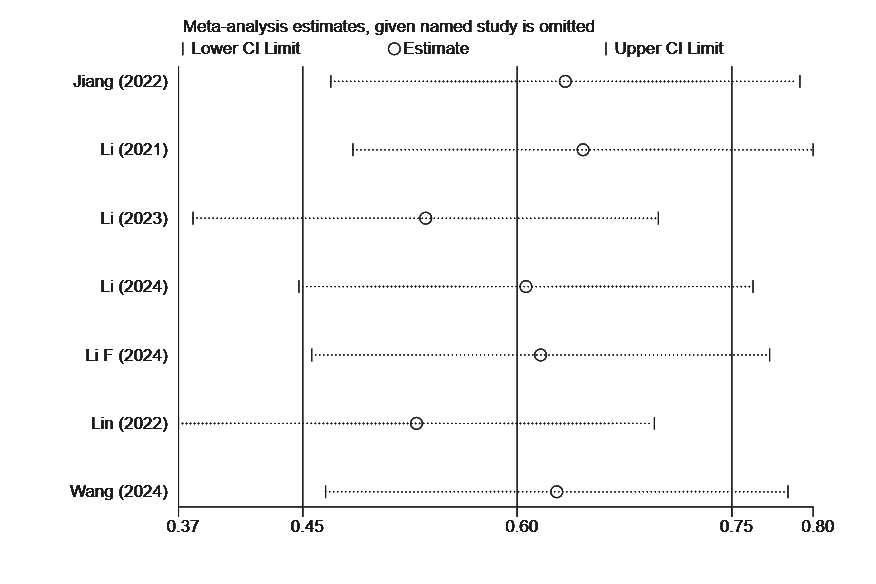


**Supplemental Figure 10. Sensitivity analysis of endometrial thickness.**

.
